# Obesity, knee osteoarthritis and knee arthroplasty: a review

**DOI:** 10.1186/2052-1847-5-25

**Published:** 2013-12-04

**Authors:** Saif Salih, Paul Sutton

**Affiliations:** 1 Department of Trauma and Orthopaedics, Northern General Hospital, Herries Rd, Sheffield S5 7 AU, UK

**Keywords:** Obesity, Knee osteoarthritis, Knee arthroplasty

## Abstract

The incidence of obesity is rising worldwide. Obesity is a risk factor for developing osteoarthritis in the knee. Obesity and knee osteoarthritis are independently disabling conditions and in combination pose difficult therapeutic challenges. This review will discuss obesity, osteoarthritis, and the problems associated with knee osteoarthritis in an obese population. Treatment options including surgery and its success will be discussed.

## Introduction

### Obesity

Obesity is defined by the World Health Organisation (WHO) as a body mass index (BMI) of greater than 30 kg/m^2^ that may impair a person’s health [1]. A BMI between 25 kg/m^2^ and 30 kg/m^2^ is defined overweight. It is important to note the WHO includes in its definition that this excess weight is caused by adipose tissue. Athletic muscular individuals may possess a BMI greater than 30 kg/m^2^ but would not be defined as obese. In England, the National Institute for Clinical Excellence (NICE) recommends the use of waist circumference (greater than 102 cm in males and 88 cm in females) in conjunction with BMI to define obesity [2].

The cause of obesity has been a subject for debate for over a century and remains controversial [3]. Some contend that obesity is caused by overeating alone, and a net positive calorific intake leads to weight gain [4]. An alternative hypothesis is that the type of calorific intake is important and that the increase in refined sugars as a proportion of our diet is responsible for the rise in obesity [5]. Others argue that it is not merely the individual’s dietary intake, but host factors such as abnormal biochemical processing, or hormonal factors contribute [3]. Research on how diet affects body weight is difficult to control and therefore much of the evidence is retrospective [6]. Although there is a need for well controlled prospective studies to guide future management of obesity and public health policy there is without doubt at present a significant disease burden as a direct result of obesity [3].

### Epidemiology

The WHO estimates that that the prevalence of obesity has more than doubled since 1980, with more 10% of the world’s population being defined as obese [1]. In England up to a quarter of the population are obese [7], with a slightly higher prevalence in women compared to men [7]. This is not unique to the population of England. In 18 out 27 European Union member states over 50% of adults are overweight or obese. Similarly one third of adults in the United States, and China are obese.

### Health effects

Overweight and obesity are the fifth leading risk for global deaths and account for an estimated 44% of the diabetes burden, 23% of the ischaemic heart disease burden and between 7% and 41% of cancer burdens [1]. There is a clear link between Osteoarthritis (OA) and obesity, which will be discussed in more detail within this review.

## Review

### Osteoarthritis

OA may be defined as a condition characterised by progressive loss of articular cartilage within a joint resulting in pain [8]. It had previously been classed as a non-inflammatory joint degeneration but there is increasing evidence that the Greek etymology (osteo- bone, arthr- joint, itis- inflammation) is a more accurate reflection of the pathophysiology [9].

OA may be classified as primary or secondary [10]. Primary OA appears to have a genetic basis but no other underlying pre-determinants. Secondary OA may develop as a consequence of multiple causes (see Table 1). There is a greater prevalence of OA between siblings and especially identical twins [11]. Frizzle related protein 3 and Asporin have been identified from large genetic studies as potentially important genes contributing to OA [12].

**Table 1:** Causes of secondary OA

	
Trauma	
Infection	
Deformity	Secondary to developmental hip dysplasia or Perthes’ disease.
Avascular necrosis	
Other medical causes	Renal disease, Sickle cell disease, Haemophilia, Wilsons, Haemochromatosis, Endocrine
Skeletal dysplasia	

OA is frequently described to patients as ‘wear and tear’ that occurs with age; however, there are subtle differences between the changes in articular cartilage that occur with age and those seen in OA [9]. The hyaline cartilage that lines synovial joints consists of chondrocytes and an extracellular matrix [13]. A normal extracellular matrix is 75% water held by long proteoglycan chains. With age these chains shorten, the water content, and the number of chondrocytes decrease. In OA there is an initial increase in water content and chondrocyte number and it is postulated that this is a chondrocyte healing response to abnormal stresses [14]. With progressive disease, chondrocyte numbers and proteoglycan content fall, as does water content. This results in further damage to the articular cartilage and the release of chondral debris and inflammatory mediators. This triggers the innate immune system and starts a chronic inflammatory cycle that further damages the joint [9]. The inflammatory mediators present in osteoarthritic joints are similar to those found in rheumatoid joints but present at lower concentrations.

### Epidemiology

OA affects roughly 8 million people in the United Kingdom and approximately 27 million people in the United States [15]. The prevalence of OA increases with age. The presence of radiological changes of OA in many joints also increases with age. The reporting of pain increases with increasing severity of radiographic changes [16] but this does not mean radiographic changes equate to pain. In one study half of men and a fifth of women with severe radiographic changes in their hip or knee did not report pain [17]. The presence of osteophytes on a skyline view of the knee appears most predictive of knee symptoms.

### Knee OA

The knee joint is commonly affected by OA. The prevalence of knee OA is rising and this may be, at least in part, due to the rising prevalence of obesity and an ageing population [18]. The Dutch cohort from Zoetemeer showed increasing radiographic knee OA with age with over half of women and a quarter of men having evidence of disease [19]. There was no significant difference between the left and right knee. Data from the Framingham cohort has shown self-reported knee pain has tripled in men and doubled in women over thirty years [18]. This disease burden appears to be increasing worldwide. The prevalence of knee pain and OA in the Indian subcontinent is approximately 35% [20]. The prevalence of knee pain in the same ethnic group living in England is much greater but the rate of OA within this group is very similar. The increase in knee pain may be attributable to an unexplained increase in rheumatoid arthritis [21].

### OA and obesity

Obesity is a recognised risk factor for developing knee OA [22]. Several large studies have directly correlated obesity with the knee OA [22]–[25]. A meta-analysis of these demonstrated that there is an almost threefold increase in the risk of developing OA within an obese or overweight population [25]. This analysis also demonstrated that the risk of developing OA increased in proportion with increasing weight. Of all the studies meeting the inclusion criteria in this meta-analysis only one did not show a statistically significant increase in prevalence of OA with increasing BMI [24]. However, this study did show a three-fold increase in the relative risk of developing knee pain over a three-year period with obesity.

In identical twins the risk of developing radiographic changes at the patella-femoral and tibio-femoral joints increases with weight [23]. For every kilogram of extra weight there is a 9-13% increased risk of developing symptomatic OA. On average twins with radiographic signs of tibio-femoral OA were 2.93 kg heavier and those with patella-femoral disease were 3.50 kg heavier. The patella-femoral joint appears to be particularly susceptible to radiographic changes with increased cartilage loss with increasing BMI even in asymptomatic women [26].

The mechanical theory for the increased prevalence of OA in obesity proposes that the extra force across a joint from increased body mass is the cause, however this theory is unlikely to explain the increased prevalence of OA found in the hands of those with a raised BMI [23]. Obesity is part of the diagnostic criteria for metabolic syndrome, which is linked with a chronic low-grade pro-inflammatory state [27]. This may provide a biochemical explanation for the link between obesity and OA [14]. Adipokines have been implicated as a potential mediator of this effect. Adipokines are cytokines that are predominantly released by adipose tissue into the bloodstream [28]. Adiponectin is one of these adipokines and its concentration is increased in patients with OA, especially in obese females. However this same study demonstrated there was only a weak correlation between increased adiponectin and synovial inflammation and there was no direct correlation with articular cartilage damage [29]. In another study increased adipokines and adipokine receptors were associated with cartilage damage, but not with obesity [30]. A large study of over 1000 patients suggested that elevated adiponectin and resistin (another adipokine) were only weakly associated with OA and obesity but there was a stronger positive correlation between OA and obesity and leptin [31].

Magnetic resonance imaging studies of overweight patients are interesting. One study correlated increased body weight with a greater presence of bone marrow lesions and cartilage defects but not with knee pain [32]. Further studies demonstrated that, regardless of pre-existing radiographic severity, weight loss improved knee pain symptoms [33], but does not improve the MRI appearance of cartilage defects or bone marrow lesions [34]. These findings highlight that knee pain is subjective, as is the perception of disability secondary to knee pain [35]. Data from the Chingford cohort demonstrates that a raised BMI is an independent risk factor for self-reported knee pain regardless of radiographic severity of disease [36].

Despite evidence that weight loss can improve knee pain symptoms many patients claim that their knee pain precludes losing weight [37]. Patients with a BMI >35 kg/m^2^ are recognised to undergo surgery for OA an earlier age [38]. The reasons for this are multifactorial but in part may be that obese patients with OA are more likely to seek a surgical solution than those of a normal BMI.

### Arthroplasty in obese individuals

Associated with an ageing population and rising rates of obesity, the number of total knee arthroplasties (TKA) are rising and now surpass total hip arthroplasty (THA) [39]. It is anticipated that the number of TKA being performed in the overweight and obese is likely to continue to rise [40]. The success, failure and outcome of TKA are potentially altered in overweight or obese patients [37,38,41]–[43].

TKA in obese individuals is technically more challenging, takes longer to perform and larger exposures are required to provide surgical access. The risk of intra-operative surgical complications is higher in patients with an elevated BMI. These include higher blood transfusion requirements as a result of operative blood loss [44], difficulty in identifying anatomy leading to iatrogenic damage (in particular medial collateral ligament avulsion [45]), or mal-alignment of the prosthesis [46].

The risk of post-operative complications in obese patients is also reportedly raised with rates as high as 32% [44,45,47]. This rate is primarily caused by superficial and deep surgical site infections and post-operative venous-thromboembolic (VTE) complications [48]. For THA and TKA the risk of VTE disease increases by 1.5 for every 5 kg/m^2^ of increased BMI [49]. A meta-analysis of surgical complications showed deep infection requiring surgical debridement occurs more frequently in obese individuals with an odds ratio of 2.38 and the risk of infection is 1.9 times greater [41]. However with the data available from the included studies, the risk of revision for infection was not significantly higher in obese individuals. Interestingly this same meta-analysis suggests that there is no increased risk of intra-operative injury, or post-operative VTE complication as a result of obesity. In contrast to other studies a further confounding analysis of Italian registry data of nearly 9000 patients suggested no significant increase in intra-operative complication, or post-operative infection, VTE disease or death in the obese group [50]. In conclusion the evidence suggests a higher risk of intra and post-operative complications of TKA for OA in obese patients but there remains some controversy in this area.

### Implant survival

Data from THR wear studies suggests that implant survival is reduced in obese patients [51]. In light of this evidence it has been hypothesised that TKA would also fail more quickly in obese individuals because the increased load across the implant would increase wear. It is important to note that the mechanism of wear occurring in a TKA is different to that that is generated at a THA.

There is evidence that at five years obese patients had no increased revision rate compared with patients with a normal BMI. The authors of this study suggested that the follow-up period was too short to detect any difference or that obese patients were less active, offsetting the effect of increased loads across the joint [43]. A further study with a longer follow-up period demonstrated that at seven years the implant failure rate, as defined either clinically (Knee Society Score- KSS) or radiographically, is significantly higher in the obese population [52]. Meta-analysis shows that the overall revision rate is 1.79 times higher in an obese group of patients compared with patients with a normal BMI after five years follow up [41]. This, however, is ‘all cause’ revision and includes both revision for infection and aseptic failures. In one series the revision rate for infection was 6% [45]. It is likely that higher implant failure and revision rates in obese patients are likely to be attributable to a higher rate of wear, aseptic loosening and infection [41]. Vazquez-Vela’s et al. [53] showed that at ten years only a third of TKA in obese men aged under 60 years survived. In comparison the highest survival rate was 99.42% in non-obese women aged over 60 years.

Following TKR obese patients do report an improvement in symptoms based on the KSS [43,54,55]. Many groups report that post-operative KSS is significantly lower in an obese population, however, the level of improvement is similar to the non-obese group [45,54,55]. Several groups report that the obese are less happy with their TKA [37,42] but these patients may appear to perform worse because of other medical co-morbidities [37]. Pre-operative obesity attributed to the inability to exercise because of knee pain does not appear to resolve with surgery. Dowsey et al. [37] reported that 21% of an obese group of patients gained weight and only 14% lost weight after TKA.

The morbidly obese represent a different subclass within the overweight and obese population. These are defined as patients with a BMI >35 kg/m^2^[1]. There is evidence that this group responds differently to the group of patients who are overweight or obese (but not considered morbidly obese) to TKA.

The surgical challenges are more extreme in the morbidly obese, they are slower to recover post-operatively [54] and have a greater rate of complications [48,55,56]. With a BMI >40 kg/m^2^ patients have lower self-reported outcome scores, increased radiographic evidence of aseptic loosening and higher revision rates [48]. Patient reported outcome is worse in the morbidly obese compared to the non-morbidly obese but the morbidly obese show bigger improvements in function when compared to their pre-operative state [57] and by three years the morbidly obese appear to do as well as non-obese counterparts [54].

Interestingly survival at 80 months appears better in the morbidly obese compared with non-morbidly obese patients, but both are worse than the non-obese patients [52]. The explanation for this may be because the morbidly obese are less active than the non-morbidly obese. A further confounding effect is suggested by some studies, which point to data skewing of the whole obese population by the subgroup of the morbidly obese. Some authors claim that if the non-morbidly obese are analysed independently of the morbidly obese population they behave in a similar manner to a non-obese population in terms of outcome and implant survival [55]. In the authors’ opinion weight loss in the morbidly obese prior to knee surgery should be advised to reduce complication rate and improve long-term outcome. Bariatric surgery may be considered as a method of rapid weight loss prior to TKA and there is evidence that this improves the radiographic evidence of OA and symptoms [58]. There is however only limited evidence that this improves post-operative complication rates for those who undergo TKA and are morbidly obese prior to knee surgery [59].

## Conclusions

Obesity is an increasing worldwide problem and the demand for TKA in this patient group is likely to continue to increase.

Obese patients are at greater risk of peri-operative complications and the overall evidence suggests they are at increased risk of premature joint failure and revision. The overall outcome of TKA in obese patients is worse than the non-obese group; however, the absolute improvements are similar in both groups. Obese patients should not expect TKA to enable them to lose weight.

There is data to support the use of TKA in an obese population and that it can provide long term benefit, but the patients must be made aware of the increased risk of peri-operative complications, the potential for re-operation and advantages of weight loss prior to surgical treatment.

## Abbreviations

WHO: World health organisation; BMI: Body mass index; NICE: National institute for clinical excellence; OA: Osteoarthritis; TKA: Total knee arthroplasty; THA: Total hip arthroplasty; VTE: Venous thrombo-embolic; KSS: Knee society score.

## Competing interests

The authors declare that they have no competing interests.

## Authors’ contributions

SS performed the literature search and wrote the first draft of the manuscript. PS conceived the article, critically reviewed the first draft of the manuscript. Both authors have read and approved the final version of the manuscript.

## Authors’ information

SS is an orthopaedic trainee at registrar level. SS is currently undertaking a subspecialty rotation in knee arthroplasty and soft tissue knee surgery. PS is a consultant orthopaedic surgeon of over twenty years’ experience. He has a specialist interest in knee surgery.

## Pre-publication history

The pre-publication history for this paper can be accessed here:

http://www.biomedcentral.com/2052-1847/5/25/prepub
